# Marked variations in diversity and functions of gut microbiota between wild and domestic stag beetle *Dorcus Hopei Hopei*

**DOI:** 10.1186/s12866-023-03177-1

**Published:** 2024-01-18

**Authors:** Yikai Lu, Siyuan Chu, Zhiyuan Shi, Ruobing You, Haimin Chen

**Affiliations:** 1 BASIS International School Hangzhou, Hangzhou, 310020 Zhejiang China; 2 Key Laboratory of Plant Secondary Metabolism and Regulation of Zhejiang Province, College of Life Sciences and Medicine, Zhejiang Sci-Tech University, Hangzhou, 310018 Zhejiang China

**Keywords:** *Dorcus Hopei Hopei*, Habitat ecology, Gut microbiota, 16S rRNA gene sequencing, Bioinformatics

## Abstract

**Background:**

Although stag beetles are a popular saprophytic insect, their gut microbiome has been poorly studied. Here, 16 S rRNA gene sequencing was employed to reveal the gut microbiota composition and functional variations between wild and domestic *Dorcus hopei hopei* (*Dhh*) larval individuals.

**Results:**

The results indicated a significant difference between the wild and domestic *Dhh* gut microbiota., the domestic *Dhh* individuals contained more gut microbial taxa (e.g. genera *Ralstonia* and *Methyloversatilis)* with xenobiotic degrading functions. The wild *Dhh* possesses gut microbiota compositions (e.g. *Turicibacter* and *Tyzzerella* ) more appropriate for energy metabolism and potential growth. This study furthermore assigned all *Dhh* individuals by size into groups for data analysis; which indicated limited disparities between the gut microbiota of different-sized *D. hopei hopei* larvae.

**Conclusion:**

The outcome of this study illustrated that there exists a significant discrepancy in gut microbiota composition between wild and domestic *Dhh* larvae. In addition, the assemblage of gut microbiome in *Dhh* was primarily attributed to environmental influences instead of individual differences such as developmental potential or size. These findings will provide a valuable theoretical foundation for the protection of wild saprophytic insects and the potential utilization of the insect-associated intestinal microbiome in the future.

**Supplementary Information:**

The online version contains supplementary material available at 10.1186/s12866-023-03177-1.

## Background

Insects, as the most diverse and abundant class of animals, thrive in a wide range of habitats and utilize various substrates [1, 2]. In the long-term process of evolution, insects and gut microbiota have established an interdependent symbiotic relationship [3]. Previously, numerous studies have demonstrated that diverse microbes colonizing the insects gut, which played integral roles in their hosts, such as affecting the host metabolism, promoting efficient digestion, aiding the defense and detoxification ability [4–11]. Associations between symbiotic microorganisms and insects are common. For example, the gut bacterial communities found in the camellia weevil *Curculio chinensis* exhibit consistency with a potential microbial role in the detoxification of the defensive chemicals produced by Camellia trees [11]. The gut microbiota of Cerambycidae species offered a potential function in assisting with degrading lignocellulose [12].

**Fig. 1:**
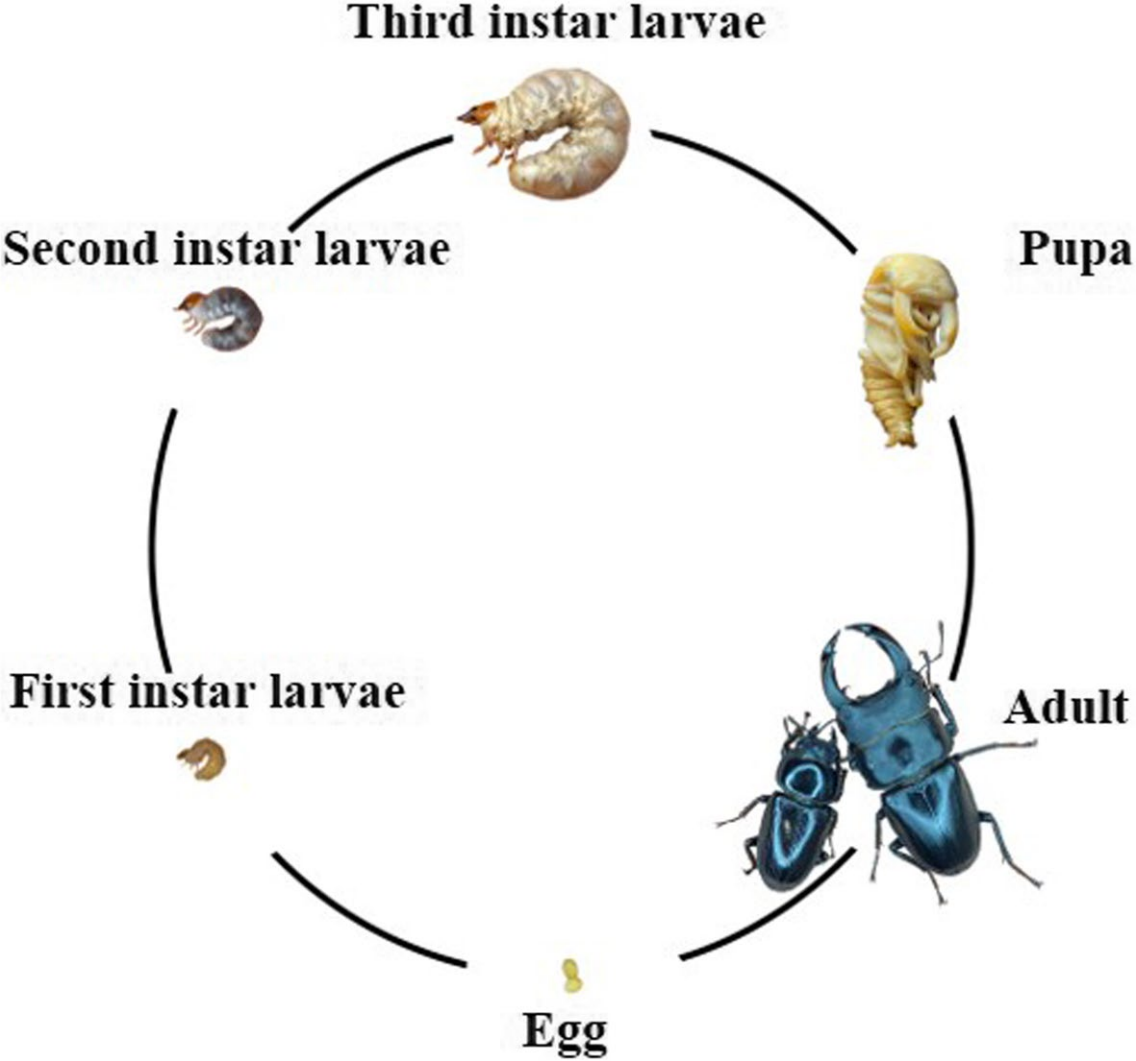
Overview of the development stages of *Dorcus hopei hopei*

*Dorcus* is a genus of the Lucanini tribe, belonging to the Lucaninae subfamily and the Lucanidae family, commonly found in China, Japan, Korea, and other East Asian countries. Currently, little research is done on the *Dorcus hopei* species, including the entire *Dorcus* genus. *D. hopei hopei* (*Dhh* in short, AKA the Giant Chinese Stag Beetle, Fig. 1), one subspecies of *D. hopei*, is predominantly concentrated in southern mainland China’s broad-leaf forests at an altitude of approximately 200–2000 m, following a wide distribution in East Asia [13]. It is one of the most abundant subspecies in the *Dorcus* genus and one of the most common stag beetles in mainland China.

The larvae are exceptionally efficient in the decomposition of timber and fungus, while the adults are important consumers of plant exudate. *Dhh* plays a vital role in the decomposition of dead wood and the carbon cycle and acts as a critical species in the maintenance of healthy forest flora. Although *Dhh* was still rarely studied, *D. hopei binodulosus* (another subspecies of *D. hopei* [14, 15]) was proven to function during nitrogen fixation [16]. Termite gut microbiota has shown to play a vital role in delignification including the *Oryctes rhinoceros* beetle, known for its consumption in rotten wood., The gut microbiota composition of the latter is similar to the gut microbiota of termites [17–19]. The gut microbiota of *O. rhinoceros* beetle may generate cellobiase to help the host cellulose degradation process [19]. Similarly, the wood-feeding beetle *Odontotaenius disjunctus* harbors a distinct fiber-associated microbiome, like the termite [20]. These results may suggest that the gut microbiota of wood-feeding insects such as *Dhh* might play a similar role.

Furthermore, the aim is to outline distinct differences between the gut microbiota composition of wild and domestic *Dhh* larval individuals. The study compared the gut microbiota disparities between different-sized larval *Dhh* to establish relationships between larval health, larval size, and specific microbial composition. These results could provide valuable insights toward future studies for the biochemistry field and the microbial conditions of stag beetles in the *Dorcus* genus. The present study is the first experimental investigation of the gut microbiota functioning and composition of the *Dorcu*s Stag Beetle.

## Results

### Sequencing information of gut microbiota in wild and domestic *Dorcus hopei hopei*

**Fig. 2:**
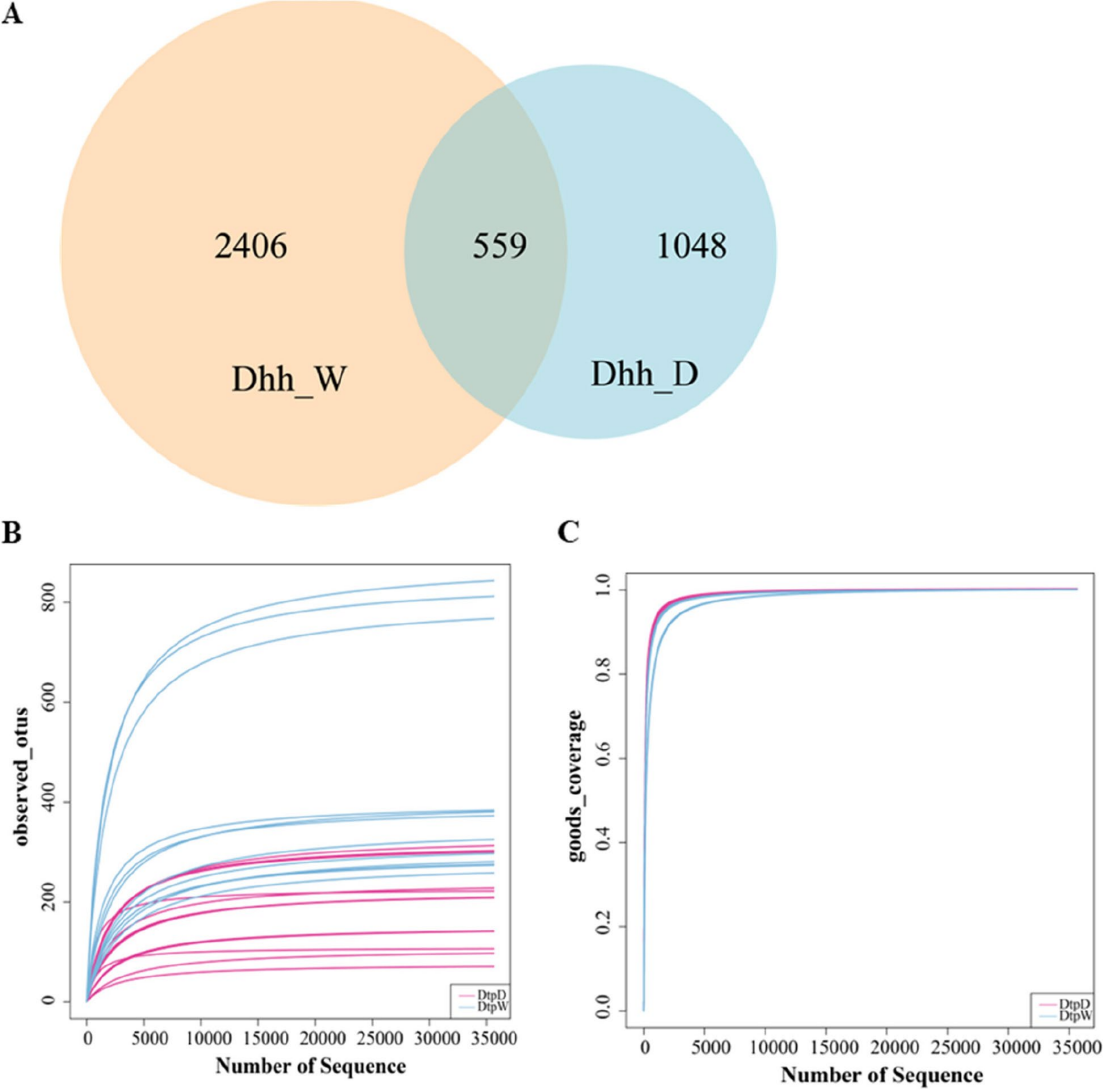
Sequencing Information of gut microbiota in wild and domestic *Dorcus hopei hopei.* **A** Venn diagram of ASVs distribution, (**B**) rarefaction curve of observed_OTUs, (**C**) rarefaction curve of goods coverage

A total of 25 samples, including 13 domestic insect gut samples and 12 wild insect gut samples, were sequenced. A total of 1,888,381 raw tages and 1,667,599 valid tages (Table S1) were obtained from 16 S rDNA amplicon sequencing. A total of 4,013 amplicon sequence variants (ASVs) were clustered, in which 33 phyla, 92 classes, 205 orders, 331 families, 732 genera, and 1,072 species were identified. The number of ASVs varied from 74 to 848 per sample, with an average of 316 ASVs per sample observed. Among the 4,013 ASVs, 2,406 ASVs were solely found in the intestinal content of *Dhh*_W, 1,048 ASVs in the intestinal content of *Dhh_*D, and 559 ASVs were shared by two *Dhh* groups (Fig. 2A). Rarefaction curves plotted from observed OTUs have reached a plateau with a high Good’s coverage index (> 99%) that indicated that the sequence depth was able to represent the majority of the ASVs present in all the 25 fecal samples (Fig. 2B, C).

### Intestinal bacterial alpha and beta diversity

**Fig. 3:**
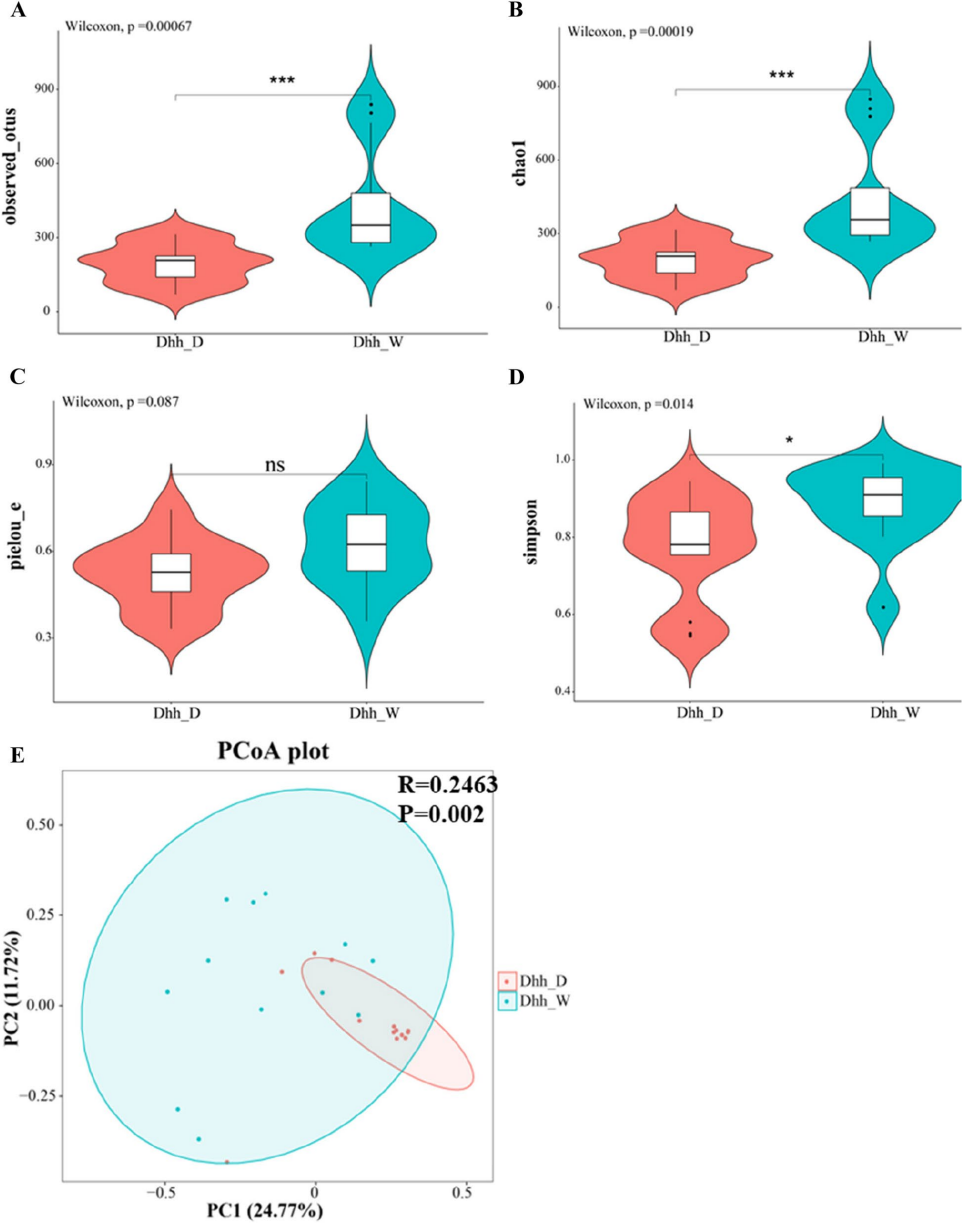
Violin plots of alpha diversity and PCoA analysis between the Wild and Domestic *Dhh* groups. **A** observed_otus, (**B**) chao1, (**C**) pielou_e, (**D**) Simpson’s index, (**E**) Principal coordinates analysis (PcoA) and Adonis analysis revealed the differences between the Wild and Domestic groups

The alpha diversity index indicated differences in gut microbiota flora between wild and domestic *Dhh* groups. According to the observed OTUs and Chao1 index, the wild group is enriched with significantly greater numbers of gut microbiota species compared to the domestic group (Fig. 3A, B). Pielou_e index illustrated that the wild group is slightly more even than the domestic group. (Fig. 3C). The Simpson index showed that the wild group had a higher value (Fig. 3D). The two indexes above showed that the diversity of the microbial communities for the wild group was higher.

Similarity analysis (ANOSIM) at ASV level was based on the Bray-Curtis dissimilarity algorithm to explore the overall difference among wild and domestic *Dhh* groups (*R* = 0.2463, *p* = 0.002). Principal coordinates analysis (PCoA) based on the Bray-Curtis distance algorithm was used to compare the community similarities between wild and domestic *Dhh* groups. The PCoA scatter plot showed that the samples of *Dhh*_D were relatively more gathered, compared to the samples of *Dhh*_W, and the two groups didn’t separate thoroughly (Fig. 3E).

### Characteristics comparison of the gut microbiota between wild and domestic *dhh*

**Fig. 4:**
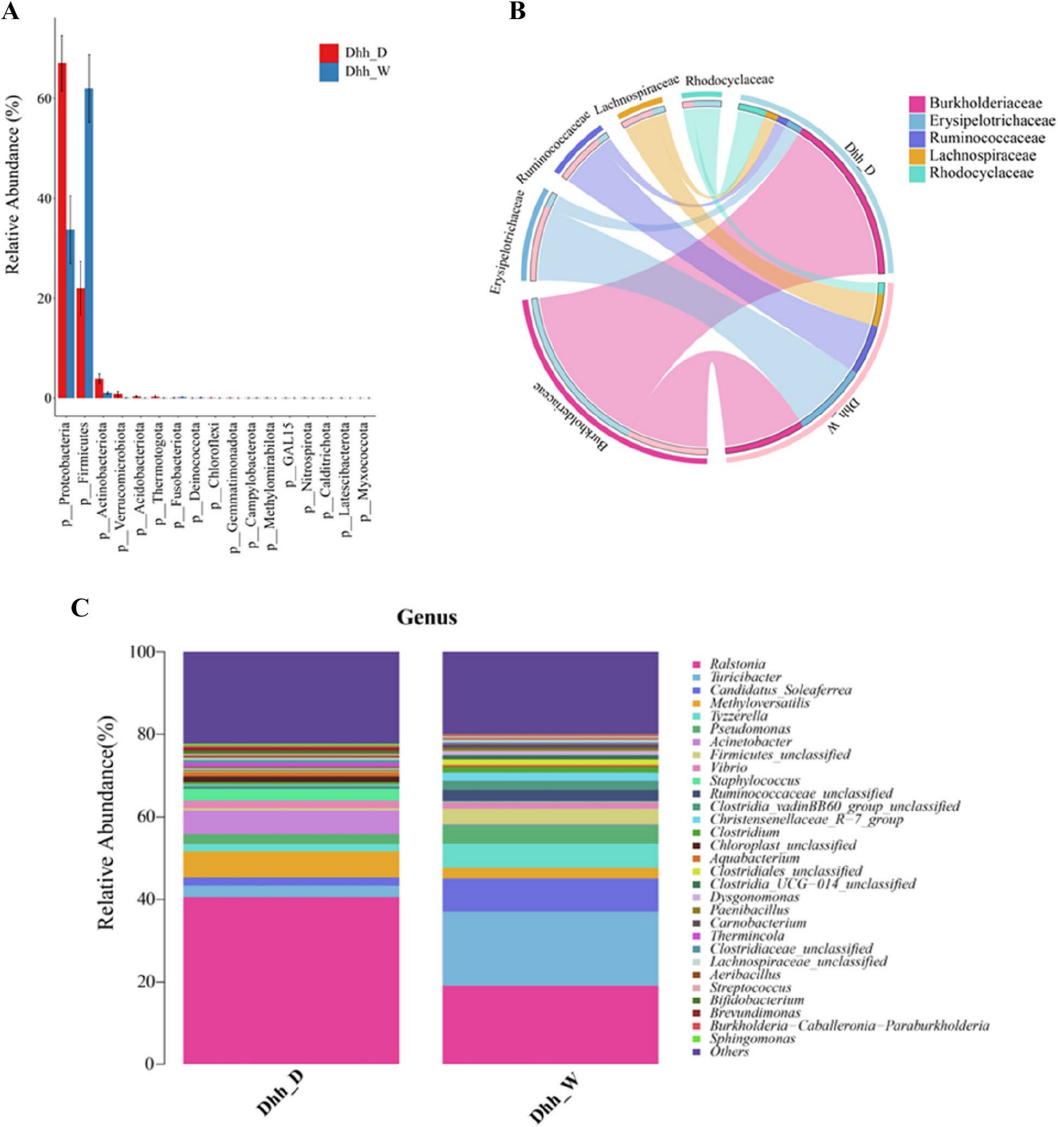
Difference between wild and domestic *Dhh *larvae feces. **A** Bar chart depicting the RA of the major detected phyla in the Wild and Domestic groups. **B** Circos plot depicting the top 5 families of the overall sample & their RA distribution in the wild and domestic groups. **C** Top genera in the overall sample & their RA distribution in the Wild and Domestic groups

The wild *Dhh* fecal microbiotas’ majority of abundance was occupied by three phyla: Firmicutes, with a relative abundance (RA) of 24.56–91.36% (mean RA: 62.02%); Proteobacteria, with a RA of 4.28–58.57% (mean RA: 33.71%); and Bacteroidetes, with a RA of 0.47–6.39% (meanRA:1.78%) (Fig. 4A). In the studied wild *Dhh*, Erysipelotrichaceae was the predominant family within the phylum Firmicutes, with Ruminococcaceae being the second-most dominant family belonging to Firmicutes. These two families are among the top 5 most abundant families in the wild and the overall sample (Fig. 4B). Other phyla observed in low abundance include Fusobacteriota (mean RA: 0.18%) and Actinobacteriota (mean RA: 1.02%), in which Fusobacteriota was almost exclusively seen in wild *Dhh* fecal samples. In the domestic *Dhh* samples, the most dominant phylum was Proteobacteria, with a RA of 28.38–89.80% (mean RA: 67.08%), followed by Firmicutes, with a RA of 2.56–67.07% (mean RA: 22.00%); and Actinobacteriota, with a RA of 0.25–10.97% (mean RA: 3.89%). Although at low abundances, multiple trace phyla expressed by domestic *Dhh* microbiota are almost exclusive to domestic samples, including Methylomirabilota (mean RA: 0.03%) and Desulfobacterota (mean RA: 0.21%) (Fig. 4A).

The RA of Firmicutes was higher in wild *Dhh* compared to that of domestic *Dhh* (Wilcoxon test, *P* < 0.01), whilst the RA of Proteobacteria in wild *Dhh* was lower compared to that of domestic *Dhh* (Wilcoxon test, *P* < 0.05). Bacteroidetes RA showed no significant difference between the two *Dhh* groups (Wilcoxon test, *P* = 0.87). Burkholderiaceae, belonging to the phylum Proteobacteria, was the predominant family in both wild and domestic *Dhh* samples and did express significant differences between its abundance in the two *Dhh* groups. Its RA for the combined sample reached an impressive 30.71% on average, with a mean RA of 41.02% and 19.54% for the domestic and wild samples, respectively. Another family belonging to the Proteobacteria phylum, Rhodocyclaceae, is among the top 5 most abundant bacterial families for the overall & separate samples (Fig. 4B).

From the genus levels, *Ralstonia* was the predominant genus in the combined sample and both individual *Dhh* groups (mean RA: 67.08%). However, there were still stark differences in terms of RA of *Ralstonia* between the wild and domestic samples, with *Ralstonia*’s RA being significantly higher in domestic *Dhh* compared to wild *Dhh* (40.63% vs. 19.17% respectively). The second overall most abundant genus was *Turicibacter* (mean RA: 9.99); its mean RA was only 2.75% in domestic *Dhh* compared to the 17.84% in the wild sample. The abundance of other genera, such as *Tyzzerella*, *Methyloversatilis*, and (Candidatus) *Soleaferrea*, also demonstrated a significant difference in wild *Dhh* compared to domestic *Dhh* (Wilcoxon test, *P* < 0.05). These three genera, combined with *Ralstonia* and *Turicibacter*, constitute the five dominant genera in terms of relative abundance in the overall sample (Fig. 4 C).

**Fig. 5:**
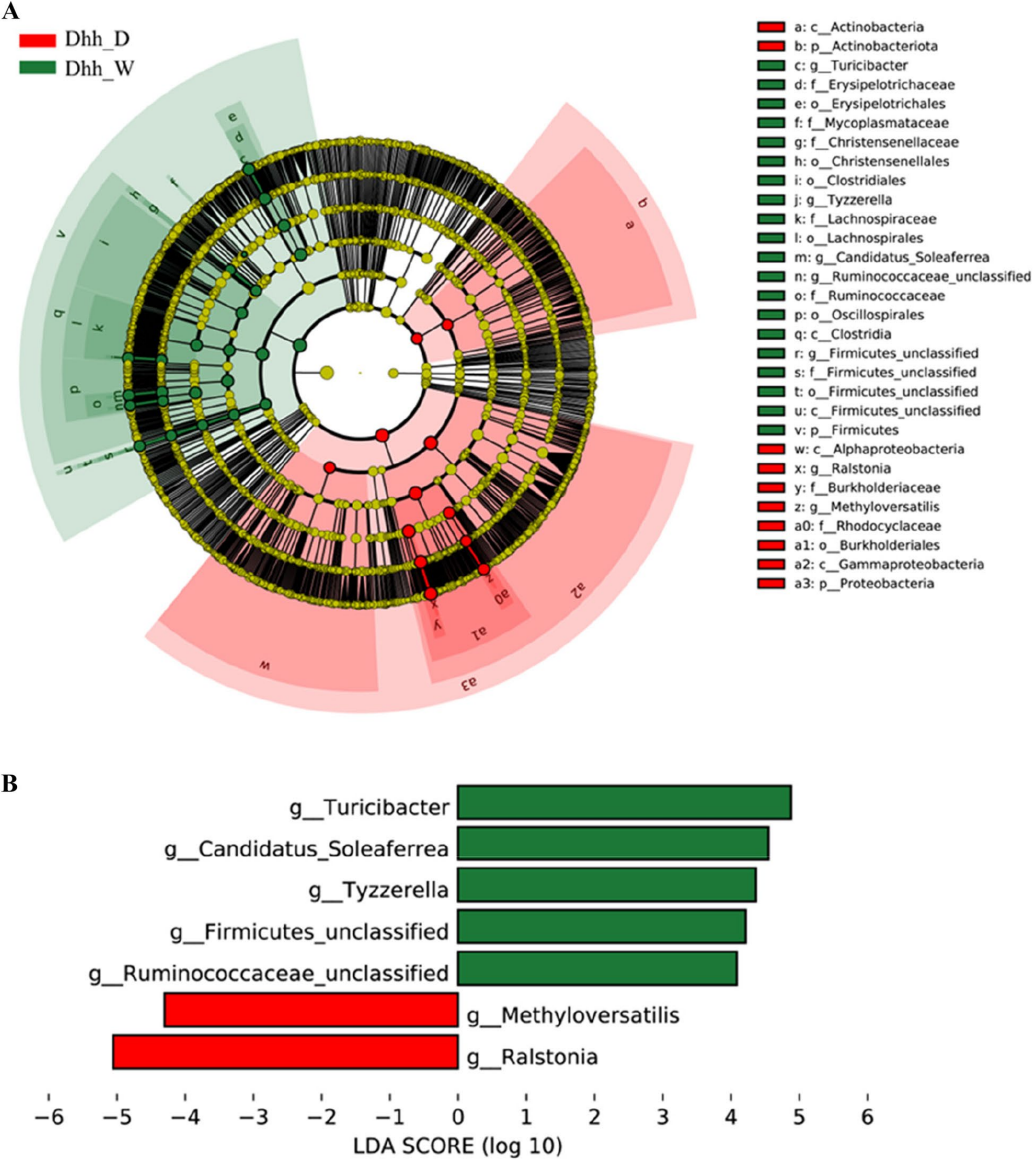
Bacterial taxon differences between the wild and domestic *Dhh *larvae feces microbiota by LEfSe in genus level. **A** Linear Discriminant Analysis Efect Size (LEfSe) cladogram. **B** LEfSe histogram. The threshold for the logarithmic discriminant analysis (LDA) score was 4

The Linear discriminant analysis effect size (LEfSe) analysis further identified numerous taxonomic clades that demonstrate statistically significant differences between wild and domestic *Dhh* (Fig. 5A). A Cladogram of the phylum to species was drawn to fully understand the distribution of different taxa at various taxonomic levels. It was found that the bacteria in two phyla (i.e., Actinobacteria and Proteobacteria), three classes (i.e., Actinobacteria, Gammaproteobacteria, and Alphaproteobacteria), and one order (i.e., Burkholderiales) were more significantly abundant in *Dhh*_D. Bacteria in one phylum (i.e., Firmicutes), two classes (i.e., Clostridia and Firmicutes_unclassified), six orders (i.e., Erysipelptrichales, Christensenellales, Clostridiales, Lachnospirales, Oscillospirales and Firmicutes_unclassified). The logarithmic discriminant analysis (LDA) results showed that the genus level biomarkers in the gut microbiota of domestic *Dhh* group were *Ralstonia*, and *Methyloversatilis*, while the enriched genera of wild *Dhh* group were *Turicibacter*, *Gandidatus_Soleaferrea, Tyzzerella*, and two unclassified genera of Firmicutes and Ruminococcaceae respectively (Fig. 5B).

### Network analysis of wild and domestic *dhh* gut microbiota

**Fig. 6:**
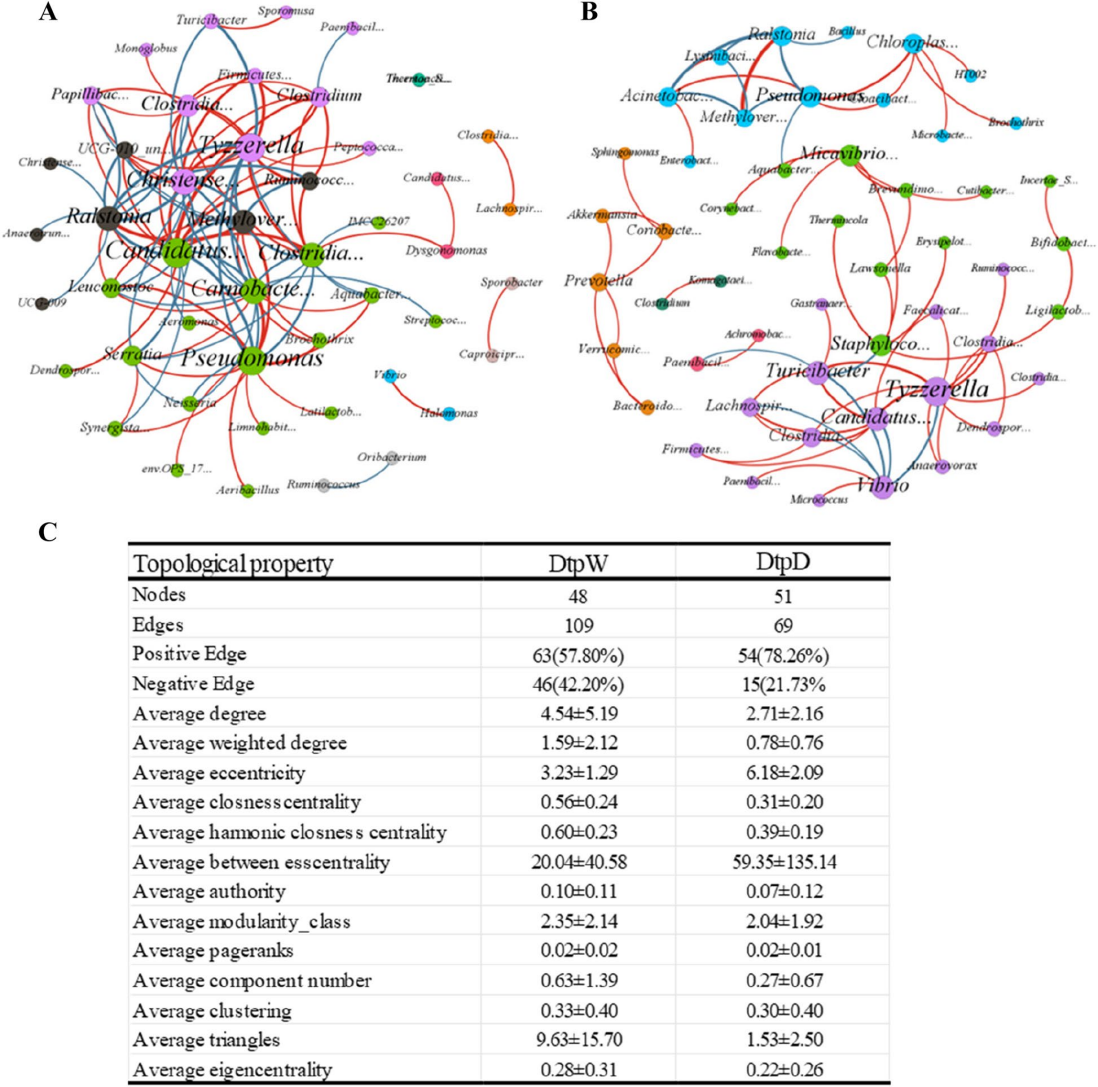
Interaction network of microbiota at the genus level in (**A**) wild and (**B**) domestic Dhh groups. The edge colors represent positive (red) and negative (blue) correlations. The size of each genus is positively correlated with its importance, and the thicker line indicates a stronger correlation. The Only significant interactions are shown (using Sparcc, |r| > 0.2; *P* < 0.05). **C** Topological property of the bacterial network of Dhh_W and Dhh_D

Interaction network analysis of the bacterial microbiome in each group was used to select the key species and to unveil the relationship of the microbiome in the community (Fig. 6). The network analysis found 48 nodes and 109 edges in Dhh_W and 51 nodes and 69 edges in Dhh_D. Compared to the Dhh_D, the edges, and average triangles presented much higher, which indicated that a more complex network was observed in Dhh_W. Also, the average weighted degree was much higher in Dhh_W, which probably indicates that the nodes in the network of Dhh_W were more important than those of Dhh_D. More network topological attributes are present in Fig. 6C. Furthermore, different dominant genera were observed in three Dhh groups individually (estimated by weighted degree). In Dhh_D, Tyzzerella, Ralstonia, (Candidatus) Soleaferrea, Turicibacter, and Vibrio greatly contributed to the interaction complexity. In Dhh_W, Tyzzerella, (Candidatus) Soleaferrea, Pseudomonas, Christensenellaceae_R-7_group, and Ralstonia played an important role in the complexity of community.

### Predicted functions of wild and domestic *dhh* gut microbiota

**Fig. 7:**
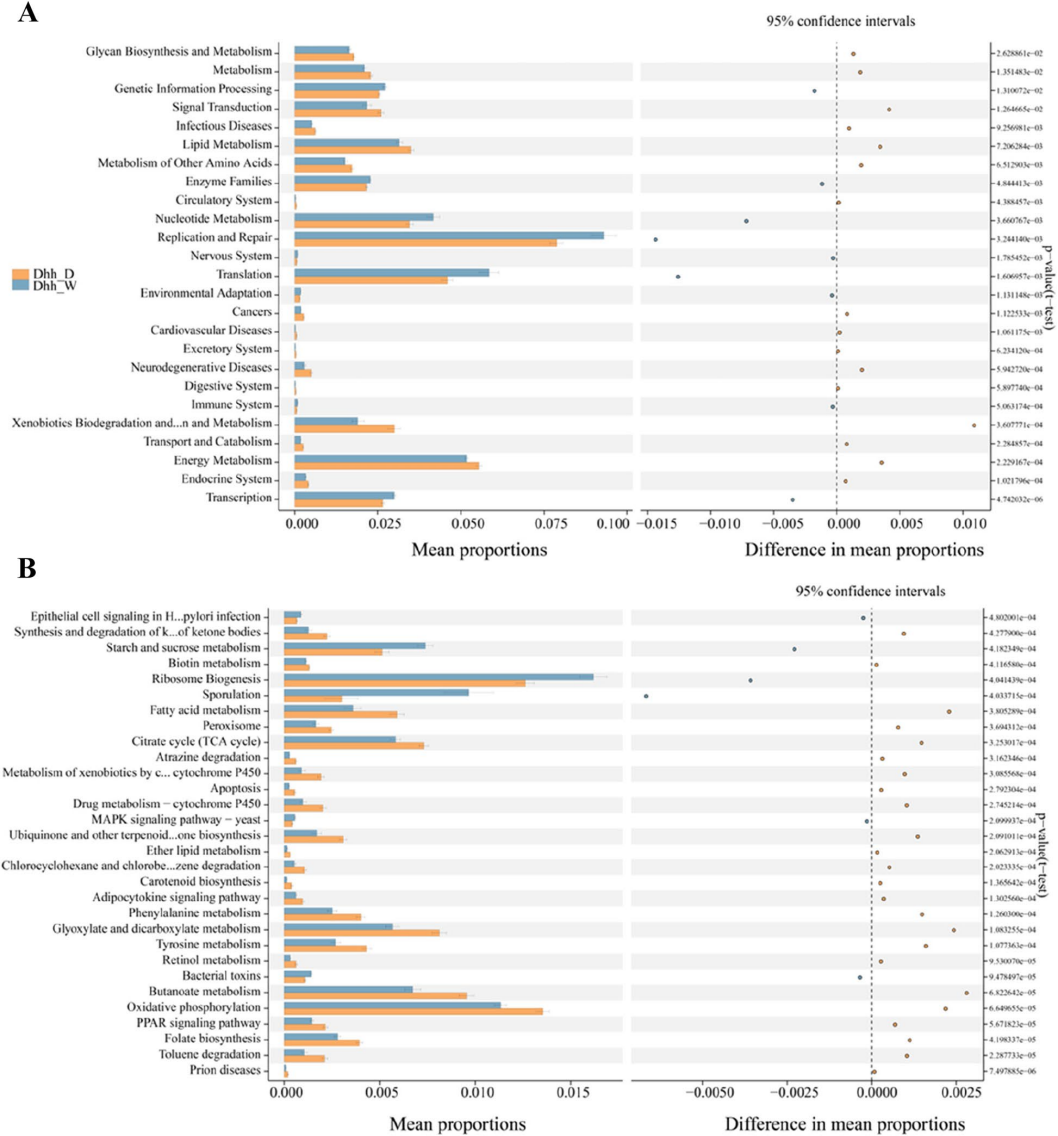
Prediction of altered KEGG pathways using PICRUSt2 analysis of the fecal microbiota for Dhh groups Domestic vs. Wild. The prediction of altered KEGG pathways in (**A**) level 2 and (**B**) level 3. Bar plots on the left side display the mean proportion of each KEGG pathway. Dot plots on the right show the differences in mean proportions between the two indicated groups. P-value was calculated using t-test

The microbiota functions were predicted using Phylogenetic Investigation of Communities by Reconstruction of Unobserved States (PICRUSt2) based on the detected 16 S rRNA gene amplicon sequences. The anticipated pathways and functions showed significant differences in microbiota functionalities between wild and domestic *Dhh*. Kyoto Encyclopedia of Genes and Genomes (KEGG) level 2 and level 3 pathways (Fig. 7), such as “Nucleotide metabolism”, “replication and repair pathways”, “environmental adaptation”, “starch and sucrose metabolism”, “ribosome biogenesis pathway”, “sporulation”, and “bacterial toxin synthesis” pathways were significantly more enriched (Student’s t-test, *P* < 0.01) in wild *Dhh*. On the other hand, “glycan biosynthesis and metabolism pathway”, “lipid metabolism pathway”, “metabolism of other amino acids”, “xenobiotics5 biodegradation and metabolism”, “energy metabolism”, “TCA cycle”, “glyoxylate and dicarboxylate metabolism”, and “oxidative phosphorylation pathways” were enriched significantly greater in domestic *Dhh* group.

### Microbiota differences of larval *dhh* of different sizes

All 4 indexes used previously for the alpha diversity of wild vs. domestic *Dhh* group, demonstrated no significance in terms of gut microbe OTUs between different *Dorcus* size groups (Figure S1, Table S2). Similarity analysis (ANOSIM) at the ASV level based on Bray-Curtis dissimilarity explored no significant difference among *Dhh*_S, *Dhh*_M, and *Dhh_*L. The PCA (*P* = 0.416) and PCoA (*p* = 0.342) analyses were used to visualize the results, which coincides with the ANOSIM analysis result (Figure S2). The bacterial community possessed a similar structure among *Dhh*_S, *Dhh*_M, and *Dhh_*L, which indicated the size of *Dhh* makes no significant difference in the gut bacterial microbiota.

At the phylum level, the overall top 5 most dominant phyla remain to be Proteobacteria, Firmicutes, Actinobacteriota, Bacteroidetes, and Cyanobacteria. The column chart depicted a significant differences in the relative abundance of the top 5 phyla in larval *Dhh* of different sizes (Figure S3A). The depicted disparities of relative genera abundance were also less pronounced compared to the wild vs. domestic analysis, with only 9 of the 718 tested genera revealing statistically significant differences for relative abundance between the three *Dhh* groups. Of the 9 genera, only *Turicibacter* was among the top 5 most dominant genera in the overall sample (Figure S3B).

Very minimal predicted pathways and functions were shown to have different levels of enrichment between the three *Dorcus* size groups. The “signaling molecules and interaction” pathway (level 2) was shown to be distinguished enriched among the three groups (Figure S4A). The “ribosome biogenesis” function (level 3) is only enriched at inferior levels in the “Small” group and the “cyanoamino acid metabolism” (level 3) is only greatly enriched in the “Large” group (Figure S4B). The “Wnt signaling pathway” was also enriched at statistically significant levels. Although significant, this pathway’s depicted deficient proportion was deemed unfit for further inspection (Figure S4C).

## Discussion

The investigation of the gut bacterial community of *Dhh_*W and *Dhh_*D was conducted through high-throughput sequencing, which was the first study of the gut bacteria of *Dhh.* Firmicutes and Proteobacteria were dominant in the gut bacteria of *Dhh_*W and *Dhh_*D, which is consistent with the prior studies in other beetles, including *Phalacrognathus muelleri* [21], *Anoplophora glabripennis* [22], *Hylobius abietis* [23], *Nicrophorus vespilloides* [24], and *Popillia japonica* [25]. Previous research has indicated that Firmicutes play a significant role in the degradation of plant carbohydrates, while Proteobacteria contribute to nitrogen fixation and metabolism [21, 26].

As a result, the alpha diversity of *Dhh*_W was higher than that of *Dhh*_D. This finding is consistent with a previous study found in species of *Bos gaurus* [27], *Mus musculus* [28]. Intestinal bacteria communities are regarded as originating from the environment and diet [29]. Therefore, the high diversity displayed in *Dhh*_W may be due to the high microbial colonization from a relatively diverse dietary intake in the wild environment. PCoA and ANOSIM analysis revealed a significant difference between *Dhh*_W and *Dhh*_D communities, suggesting that the host environment serves a crucial role in shaping intestinal bacterial structure. Previous studies demonstrated that gut bacteria communities can influence the host diet [30–33]. Although the study tried to provide *Dhh*_D with the same food source as *Dhh*_W, there may still be some unrealized differences in food composition, which may be one of the reasons for community differences.

Although differences between gut microbial communities were observed, there are also observable similarities as well. For instance, although Firmicutes were expressed with higher RA and Proteobacteria expressed at a lower RA in wild *Dhh* compared to the domestic group, the two phyla combined comprised more than 85% of the bacterial species in the domestic group and more than 95% in the wild group.

The statistics reflect the significance of these two phyla in forming larval *Dhh* gut microbiota; furthermore, the discrepancy in RA of the two major phyla among the two *Dhh* groups demonstrates the environmental and ecological impact on the Firmicutes-to-Proteobacteria ratio and overall microbiota compositional balance. The top 5 most dominant families in the overall sample are Burkholderiaceae, Erysipelotrichaceae, Ruminococcaceae, Lachnospiraceae, and Rhodocyclaceae. Three belong to Firmicutes and two to Proteobacteria, parallel to the obtained phylum-level statistics. *Ralstonia* was responsible for the abundance of Burkholderiaceae, *Turicibacter* for Erysipelotrichaceae, *Tyzzerella* for Lachnospiraceae, and *Methyloversatilis* for Rhodocyclaceae.

The family Ruminococcaceae was distinctive as its abundance was the second-most dominant family belonging to Firmicutes in wild *Dhh* groups, which some taxa in this family presumably share certain beneficial features favorable for polysaccharides biodegradation in *Dhh* gut. This speculation was supported by some general Ruminococcaceae known capacity for structural polysaccharides breakdown [34, 35] and further testified by the strong positive association between Ruminococcaceae and tetrapod herbivory [34]. The study cannot conclude that the specific function of Ruminococcaceae in *Dhh* gut microbiota will be identical to that of other tetrapod herbivores due to physiological differences, current knowledge about biochemical potentials of Ruminococcaceae does imply high probabilities of this bacterial family conducting similar roles in *Dhh* gut microbiota.

Previous studies have utilized microbial correlation network analysis to investigate the differences in microbial community structure [36–39]. Microbial correlation network analysis allows the identification of the key species in the entire network, by identifying the species with high connectivity throughout the network, and these species may have a crucial role in the structure and function of the microbial community [40]. Our study performed microbial correlation network analysis and observed several topological characteristics of the network. Specifically, the study found an increasing number of edges and higher average triangles, indicating an enhanced complexity of community in *Dhh*_W. Besides, the proportion of positive association decreased, and that of negative association increased in *Dhh*_W, which indicated a more balanced community association. The key nodes with a high degree, in the network, probably exert an essential function in maintaining gut microbiota and in exerting specific beneficial functions. Network analysis indicated that a more complex and stable bacterial community was observed in *Dhh*_W, which coincided with our anticipation. It’s probably the complex environment and relatively diverse food intake that leads to the complex intestinal bacterial community and its potential function.

Overall, although existing at different abundances, both Wild and Domestic *Dhh* group exhibited significant gut microbiota communities associated with xenobiotic degradation and metabolism. Out of the top 5 genera, the ones responsible for metabolism and plant material breakdown (*Turicibacter*, *Tyzzerella*, *Soleaferrea*) were more significantly enriched in the Wild group; genera with xenobiotic degradation abilities (*Ralsonia*, *Methyloversatilis*) were more abundant in the Domestic group, completely aligning with trends identified by PICRUSt2 results. Xenobiotics-degrading microbes’ elevated presence is potentially the direct effect of the artificial exacerbation of *Dhh* larvae’s habitat ecology.

As the most abundant genus in *Dhh_*W and *Dhh*_D, the soil-borne bacterial genus *Ralstonia* was widely regarded as a plant pathogen, causing bacterial wilt in plants [41]. *Ralstonia* includes mostly plant pathogenic bacteria capable of growing on plant oils/fatty acids, and expresses genes such as aceA1 and aceA2 that code for enzymes involved in the glyoxylate cycle [42]. This indicates that *Ralstonia* potentially provides *Dhh* with metabolites derived from previously undegradable plant lipids and aids in *Dhh* cells aerobic respiration. *Ralstonia* could utilize an array of aromatic compounds and heavy metals for energy for detoxification [43]. These compounds are primarily xenobiotic and hazardous to organism health. *Ralstonia* species are especially valuable gut microbes to form symbiotic relationships with larval *Dhh*, whose nutrition sources often experience contamination during decay. PICRUSt2 reinforced our hypothesized function of *Ralstonia* in larval *Dhh* gut microbiota. The functions of “lipid metabolism,” “xenobiotics biodegradation and metabolism,” “TCA (tricarboxylic acid) cycle,” and “glyoxylate metabolism” were all significantly enriched in the domestic group, the group of higher *Ralstonia* RA. These results indicate that the microorganisms being regarded as plant pathogens probably play a vital role in insects by improving metabolism and detoxification. Furthermore, since the rearing of domestic larvae occurred in human infrastructure, the domestic group is indeed expected to be exposed to greater levels of xenobiotics, validating PICRUSt2 data and *Ralstonia*’s predicted functions.

The genera (Candidatus) *Soleaferrea* and *Tyzzerella* are denoted to contribute to plant-matter breakdown. Both were identified as core microbiota genera of *Macrotermes falciger* (a termite species), a species sharing similar diets with *Dhh* larvae (xylophagous & mycophagous) [44]. Furthermore, *Tyzzerella* was significantly enriched in the gut microbiota of Bark beetles which served as forest damaging agents [45].

The microbiota differences between the *Dorcus* size groups (L, M, S) are of less significance, as could be seen from abundance analysis and PICRUSt2 results. One actual difference between the *Dhh* group’s gut microbiota is the *Turicibacter* RA, which depicts a descending tendency as larvae size decreases. This suggests that larger *Dhh* larvae growth was potentially attributed to reinforced metabolic capabilities and internal hormone regulation provided by *Turicibacter*. The “large” group was the most enriched, the “medium” group came second, and the “small” group was the least enriched. Overall, the patterns demonstrated by *Dhh* larvae of different sizes contrasted with the former research results, as size and microbiota composition/diversity were demonstrated to be associative in the stag beetle *Odontolabis fallacios*a [46].

This present study results could be beneficial for improving the current artificial rearing methods of *Dhh* by suggesting advantageous microbial communities for larval growth, potentially leading to modified feeding procedures that cultivate more aesthetic and healthier commercial *Dhh*. More importantly, the obtained data are beneficial for developing future environmental protection and conservation methods. One hypothesized technique to be considered is the release of artificially bred *Dhh* larvae or adults with modified microbiota profiles imitating wild individuals into designated areas to facilitate material decomposition and nutrient cycling in any required habitat, such as the insect-based agri-food waste valorization [47]. Modifying the gut microbiome reduces the released specimens’ repulsive responses to a feral environment and lowers any risks of introducing foreign gut microbes into the wild population. This could alleviate nutrient cycling issues in the specified environment with minimal contamination of the gut microbiome and overall biochemical diversity of the local species.

As this study is one of the very few research focusing on the gut microbiota of the *D. hopei* and specifically the *Dhh* subspecies, these results provided additional insights into the area of Lucanidae gut microbiota and outlined several new research orientations to be conducted for further exploration or validation of information regarding gut microbiota of *Dhh.*

## Conclusions

The present study utilized 16 S rDNA sequencing to compare the fecal bacteria of wild and domestic *Dhh* larvae and between larvae of different sizes. The relative abundance of fecal microbiota showed a notable difference between wild and domestic *Dhh*. Overall, the domestic *Dhh* individuals contained more gut microbial taxa with xenobiotic degrading functions, such as genera *Ralstonia* and *Methyloversatilis*, while the wild sample possesses gut microbiota compositions more appropriate for energy metabolism and potential growth, such as *Turicibacter* and *Tyzzerella*. On the other hand, *Dhh* larvae of different *Dorcus* size groups exhibited significantly fewer disparities in larval gut microbiota composition. This finding suggested that habitat-ecology factors, rather than individual growth potential, are responsible for the assemblage of detected distinguished microbiota patterns. These results provided preliminary insights into the gut microbiota of *Dhh* and its most common subspecies and increased our current understanding of Lucanidae microbiota composition. Results also indicated future research orientations for exploring insect gut microbiome. Furthermore, the significant enrichment of xenobiotics-degrading gut microbiota functionalities in *Dhh* larvae, especially in the domestic group, demonstrates the inherent environmental presence of factors pernicious to the survival of *Dhh* larvae. This calls for conservational actions aimed at alleviating the influence of artificial infrastructure and contaminants on local ecology and biodiversity.

## Materials and methods

### Sample collection and rearing condition

The individuals from the wild were collected in decayed timber located in Hangzhou, Zhejiang Province, China. Out of the 12 wild larvae, four specimens were from Yangjiapai Village (30° 14’ 57.0768’’ N, 120° 3’ 54.3384’’ E), four from Yuewang Temple (30°09’07.0"N, 120°13’46.9"E), three from Wenbi Mountain (30°12’34.6"N, 120°05’36.6"E), and one from Baoshi Mountain (30°15’40.0"N 120°08’38.9"E). Larvae were excavated out of the initial shelter, rotten logs in broad-leaved forests shaded area, from the range of altitude between 200 and 600 m. with proper instruments, no larvae were harmed in the process, and each health condition was examined before being accepted as a sample individual. Each individual was assigned a number; their physical data (head capsule diameter) and feces samples were obtained.

Domestic larvae were raised in captivity. The larvae were given either fermented wood flakes or bottled *Coriolus versicolor* mushroom packets, instead of rotten timber presence in the wild group. All the domestic larvae were reared in a thermal controlled environment between strict temperatures of 22–24 °C for the larval life cycle. The fungus bottles and wood flakes’ humidity remained in the good range between 55 ± 5% constantly. The fungus bottle and fermented wood flake (compressed in a bottle) were changed periodically about every 3 months to prevent food decay. Larvae were kept in a dark environment and the container remained a viable ventilation, and any other human disturbance was prohibited to eliminate confounding variables. Likewise, each individual was assigned a number, and their physical data & feces samples were obtained.

### Body data collection and grouping

The head capsule diameter was measured for each larva. The larvae were held up, and the horizontal diameter of the head capsule was measured using a caliper. The results were recorded and used for grouping. The utilization of head capsule size data instead of other features as growth-potential indicators has been justified in preceding texts. The body weight of each larva was measured using a balance. Body weight was seen as the standard for fat accumulation, which is directly associated with material processing and nutrient absorption. In the process, the larvae body surface feces and wood dust were cleaned before measuring to prevent deviation.

Two grouping plans were used in this study. The purchased and foraged individuals were isolated into “Domestic” and “Wild” *Dhh* groups (*Dhh*_D and *Dhh*_W), each including 11 and 12 *Dhh* sample individuals, respectively. The same experimental individuals were assigned into groups “Large,” “Medium,” and “Small” *Dhh* groups *(Dhh*_L, *Dhh*_M, *Dhh*_S). Each corresponds to a head capsule size of equal/over 11 mm, less than 11 mm & equal/over 9 mm, and less than 9 mm), each including 6, 10, and 6 *Dhh* sample individuals respectively. Analyses concerning the two grouping systems were separately done.

### Fecal sample collection method

The development stages overview of *Dhh* is shown in Fig. 1. The collected samples were all in the third instar larvae stage. Wild individuals were collected and installed in a container filled with the wood dust processed from the original sheltering rotten timber. Domestic individuals were taken out of their feeding medium and installed in containers. larvae were brought to the laboratory under safety and were left undisturbed for a 3-day acclimation period, in which they were monitored for any potential repulsive behaviors or health condition changes. Throughout the transportation and acclimation process, the living temperature of the larvae was controlled at 24 °C. After three days, the larvae were gathered for feces collection. The larvae’s abdomen and anal region were disinfected by wiping them with 75% alcohol-soaked gauze. The feces were then naturally excreted by the larvae individually into a sterile EP tube. Sterile forceps and scoopulas were used to remove 1 gram of feces sample from each tube and place it on a sterile foil, measuring on an analytical balance to ensure it is the precise amount. After reaffirmation, feces samples were transferred into iLongsee Stool Storage Kit (Longsee Biomedical, China), and kept in -80℃ refrigerator until DNA extraction.

### PCR amplification and 16 S rDNA sequencing

DNA from different samples was extracted using the E.Z.N.A. ® Stool DNA Kit (D4015, Omega, Inc., USA) according to the manufacturer’s instructions. Nuclear-free water was used for blank. Total DNA was eluted in 50 µL of Elution buffer and stored at -80 °C until amplification in the PCR by LC-Bio Technology Co., Ltd, Hangzhou, Zhejiang Province, China.

The V3-V4 hypervariable region of the 16 S rRNA gene was amplified using indicated primers 341 F (5’-CCTACGGGNGGCWGCAG-3’) and 805R (5’-GACTACHV GGGTATCTAATC C-3’) (Logue et al., 2015). PCR amplifications were performed in 25 µL of reaction mixture including 25 ng of template DNA, 12.5 µL of PCR Premix, 2.5 µL of each primer, and PCR-grade water. The PCR conditions go as follows: 98 °C for 30 s; 32 cycles of 98 °C for 10 s, 54 °C for 30 s, and 72 °C for 45 s; and then finally 72 °C for 10 min. PCR products were verified with agarose gel electrophoresis. Throughout the DNA extraction process, high-purity water will be used to exclude possible false-positive PCR results. The PCR products were purified by AMPure XT beads (Beckman Coulter Genomics, Danvers, MA, USA) and quantified by Qubit (Invitrogen, USA). Amplicon pools were prepared for sequencing; the amplicon library size and quantity were assessed on Agilent 2100 Bioanalyzer (Agilent, USA) and with the Library Quantification Kit for Illumina (Kapa Biosciences, Woburn, MA, USA), respectively. The libraries were sequenced on the NovaSeq PE250 platform.

### Data analysis

Samples were sequenced on an Illumina NovaSeq platform according to the manufacturer’s recommendations provided by LC-Bio. Paired-end reads were assigned to samples based on their individual barcoding and trimmed by cutting off the primer sequence. Paired-end reads were merged using FLASH. Raw reads filtering was performed under specific filtering conditions to obtain precise tags according to fqtrim (v0.94). Chimeric sequences were filtered using Vsearch (v2.3.4). After dereplication, feature tables and sequences were obtained using DADA2. 5 indices of Alpha diversity including Chao1, Observed species, Goods coverage, Shannon, and Simpson, were calculated with QIIME2 according to SILVA (release 132) classifier, and feature abundance was normalized using the relative abundance of each sample. Beta diversity were calculated by QIIME2, and the graphs were drawn by R package. BLAST was used for sequence alignment, and the feature sequences were annotated via SILVA database. Additional statistical analyses were manipulated utilizing R package (v3.5.2). Principal Coordinates Analysis (PCoA) [48] was calculated using ade4 package and Principal Components Analysis (PCA) [49] was calculated using stat package. Analysis of similarities (ANOSIM ), a non-parametric statistical test, used to test the difference between *Dhh* groups, was calculated using vegan package [50]. Log-linear discriminant analysis effect size algorithm (LEfSe) analysis was performed to identify potential taxonomic groups (LDA scores > 4.0, *p* < 0.05) that can be regarded as biomarkers for different *Dhh* groups [51]. A bacterial interaction network was performed using the Fastspar [52] with a Sparcc correlation coefficient (|correlation r)|>0.2, and *p* < 0.05), and the network map was visualized using Gephi 0.9.4. Prediction of the microbial function and pathway in KEGG database based on the 16 S rRNA sequencing data was undertaken using the PICRUSt2. And PICRUSt2 Analysis was performed using the OmicStudio Analysis at https://www.omicstudio.cn/analysis/. Other diagrams were visualized using the R package (v3.5.2). Wilcoxon rank-sum test, Kruskal-Wallis H test, or student’s t-test were employed to determine statistical significance (*P* < 0.05 is statistically significant).

### Supplementary Information

**Additional file 1: Figure S1.** Violin plots of alpha diversity among Dhh_M, Dhh_S and Dhh_L. (A)observed_otus, (B) chao1, (C) pielou_e, (D)Simpson’s index. **Figure S2.** Beta diversity among Dhh_M, Dhh_S and Dhh_L. (A) Principal Component Analysis (PCA) and (B)Principal coordinates analysis (PcoA) analysis revealed the differences among Dhh_M, Dhh_S and Dhh_L. **Figure S3.** The composition of genera among Dhh_M, Dhh_S and Dhh_L. (A) The column diagram depicted the top 5 most abundance phyla difference. (B) The stacked plot demonstrated the top 30 most abundance bacterial genera distribution. **Figure S4.** Prediction of altered KEGG pathways using PICRUSt2 analysis of the fecal microbiota for groups Dhh_M,Dhh_S and Dhh_L. The prediction of altered KEGG pathways in (A) level 2, (B) level 3 and (C) pathway. Bar plots on the left side display the mean proportion of each KEGG pathway. Dot plots on the right show the differences in mean proportions between the two indicated groups. P-value was calculated using t-test. **Table S1.** Summary of gut microbiota sequencing information of wild and domestic *Dorcus hopei hopei*. **Table S2.** Statistic analysis of alpha diversity indexs of gut microbiota among different sizes* Dhh* larval.

## Data Availability

The datasets used and/or analyzed during the current study are available from the corresponding author upon reasonable request. The raw reads for all samples used in this study have been deposited into the NCBI Sequence Read Archive (SRA) database (accession numbers: PRJNA1014221) on the website: https://dataview.ncbi.nlm.nih.gov/object/PRJNA1014221.
