# Supplementary material for: Marked variations in diversity and functions of gut microbiota between wild and domestic stag beetle Dorcus Hopei Hopei
Source: BMC Microbiol. 2024 Jan 18;24:24. doi: 10.1186/s12866-023-03177-1 (PMC10795464; doi:10.1186/s12866-023-03177-1)
Supplement: Supplementary file 1 — Additional file 1: Figure S1. Violin plots of alpha diversity among Dhh_M, Dhh_S and Dhh_L. (A)observed_otus, (B) chao1, (C) pielou_e, (D)Simpson’s index. Figure S2. Beta diversity among Dhh_M, Dhh_S and Dhh_L. (A) Principal Component Analysis (PCA) and (B)Principal coordinates analysis (PcoA) analysis revealed the differences among Dhh_M, Dhh_S and Dhh_L. Figure S3. The composition of genera among Dhh_M, Dhh_S and Dhh_L. (A) The column diagram depicted the top 5 most abundance phyla difference. (B) The stacked plot demonstrated the top 30 most abundance bacterial genera distribution. Figure S4. Prediction of altered KEGG pathways using PICRUSt2 analysis of the fecal microbiota for groups Dhh_M,Dhh_S and Dhh_L. The prediction of altered KEGG pathways in (A) level 2, (B) level 3 and (C) pathway. Bar plots on the left side display the mean proportion of each KEGG pathway. Dot plots on the right show the differences in mean proportions between the two indicated groups. P-value was calculated using t-test. Table S1. Summary of gut microbiota sequencing information of wild and domestic Dorcus hopei hopei. Table S2. Statistic analysis of alpha diversity indexs of gut microbiota among different sizes Dhh larval. [file 12866_2023_3177_MOESM1_ESM.docx]

Marked variations in diversity and functions of gut microbiota between wild and domestic stag beetle *Dorcus hopei hopei*

Yikai Lu ^1, [†](http://citeseerx.ist.psu.edu/viewdoc/summary?doi=10.1.1.361.7982)^, Siyuan Chu ^2, [†](http://citeseerx.ist.psu.edu/viewdoc/summary?doi=10.1.1.361.7982)^, Zhiyuan Shi ^1^, Ruobing You ^2^, and Haimin Chen ^2,^*

**Supplementary figures**


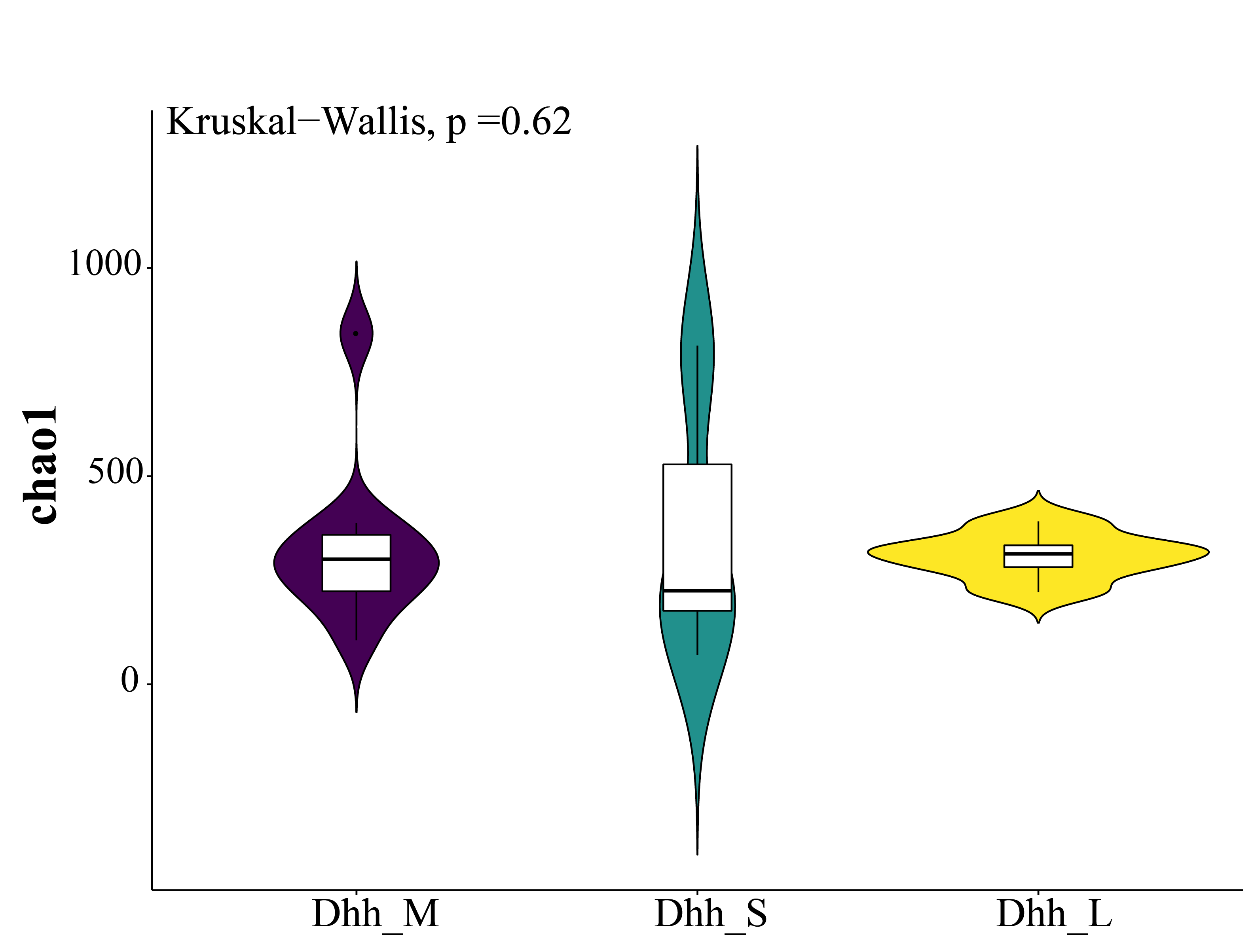

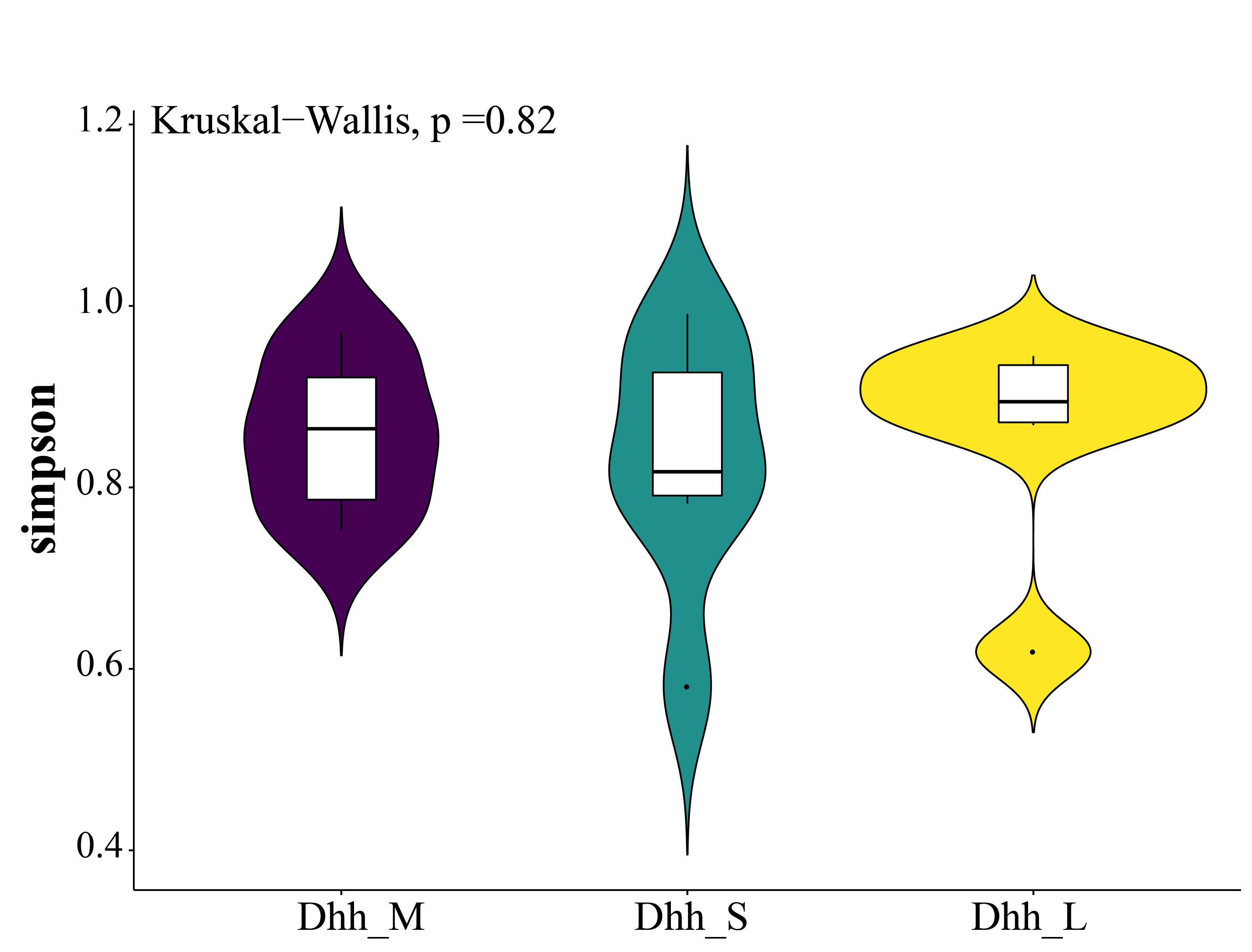

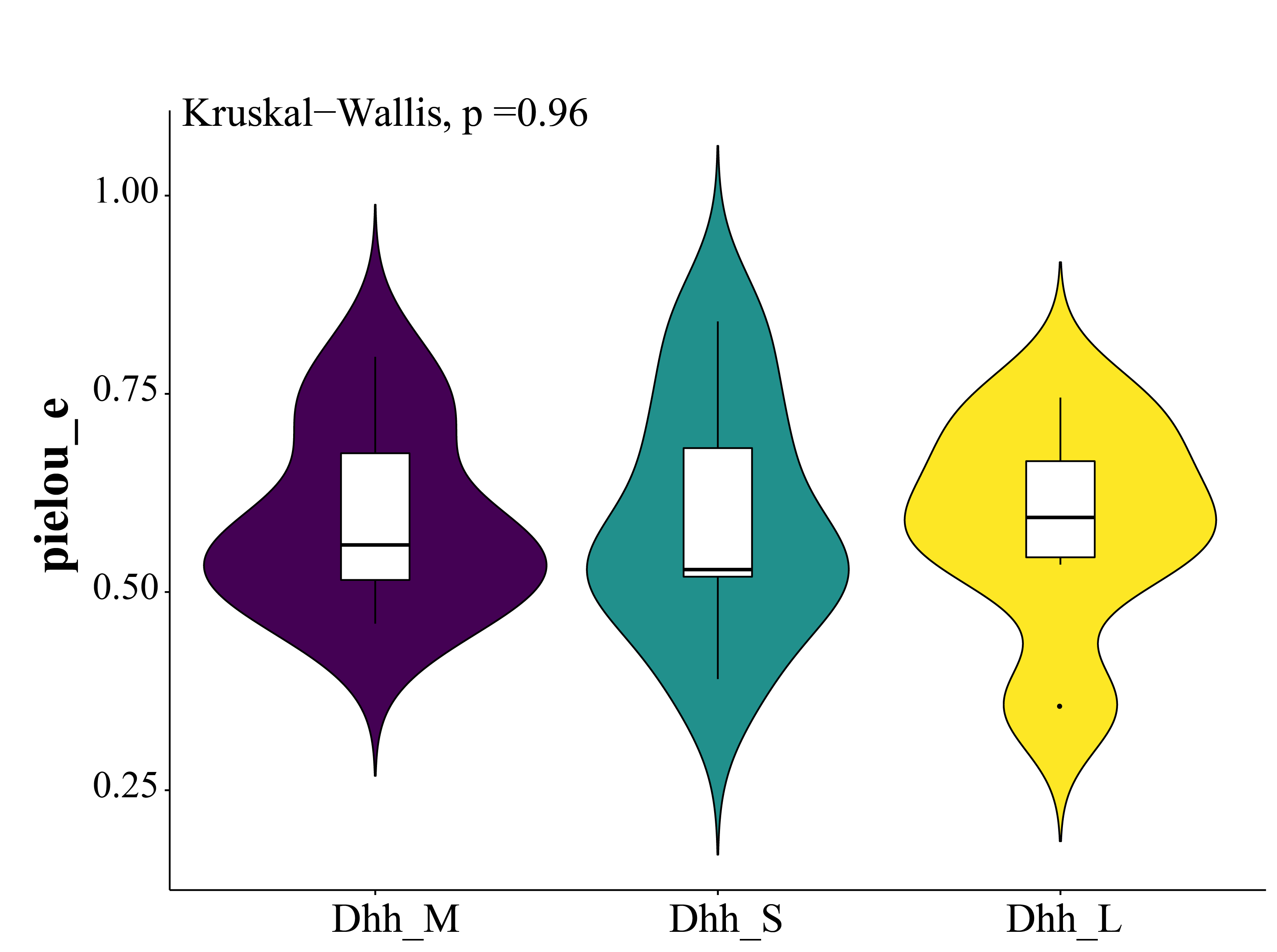

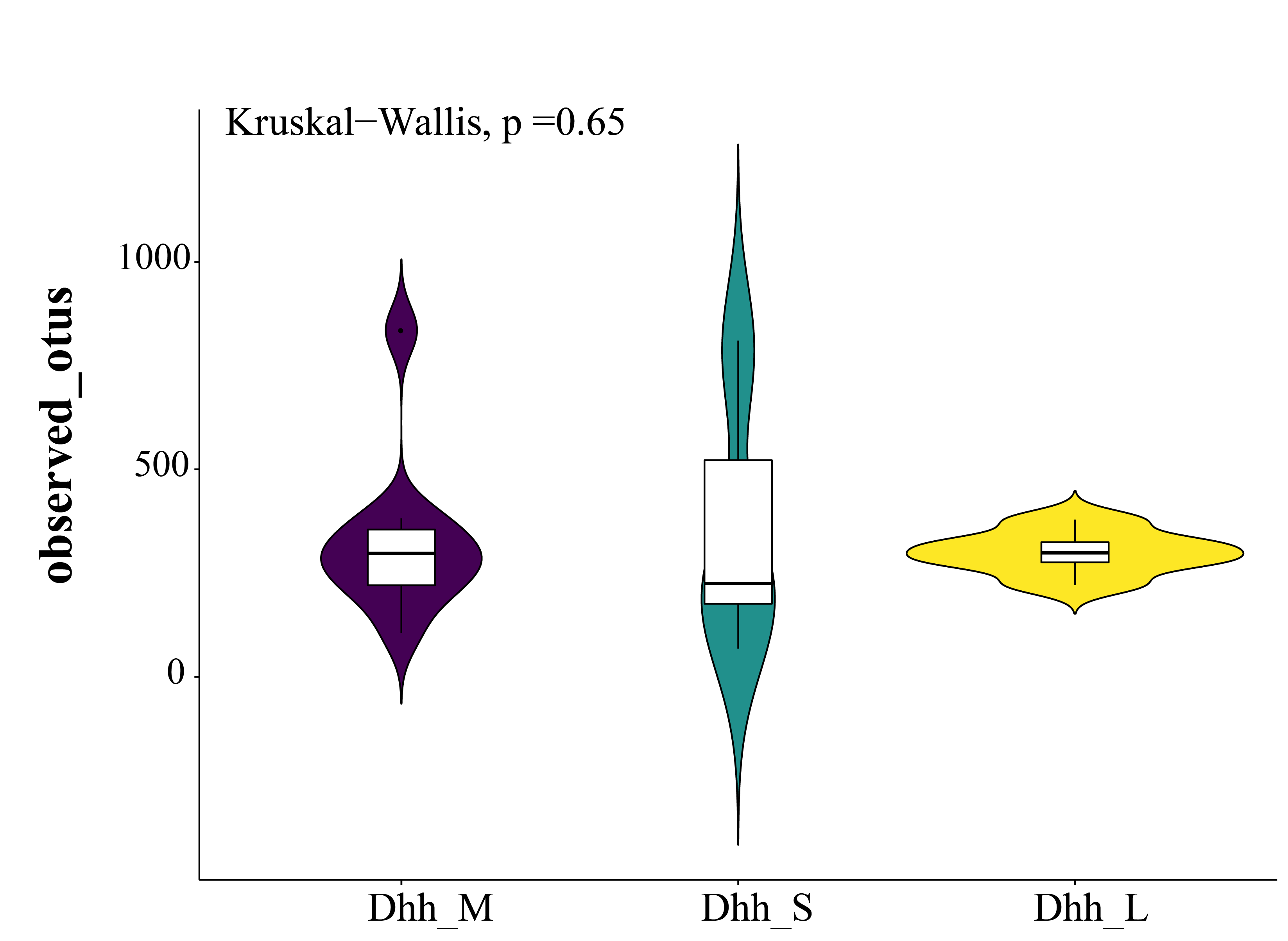


**A**

**B**

**C**

**D**

**Figure S1. Violin plots of alpha diversity among Dhh_M，Dhh_S and Dhh_L.** (A)observed_otus, (B) chao1, (C) pielou_e, (D)Simpson’s index.


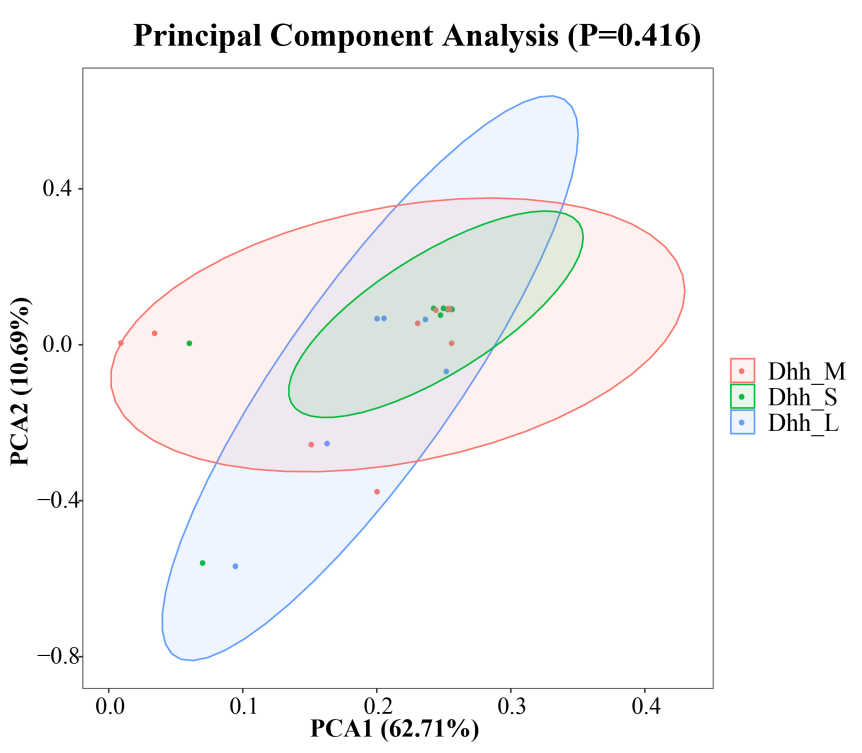

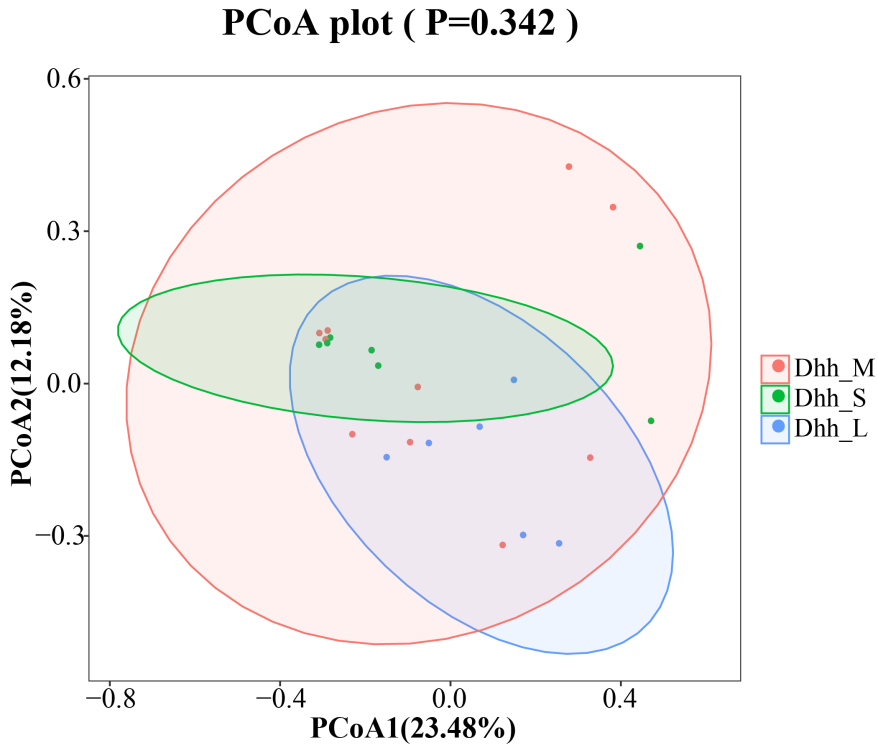


**A**

**B**

**Figure S2. Beta diversity among Dhh_M，Dhh_S and Dhh_L.** (A) Principal Component Analysis (PCA) and (B)Principal coordinates analysis (PcoA) analysis revealed the differences among Dhh_M，Dhh_S and Dhh_L.


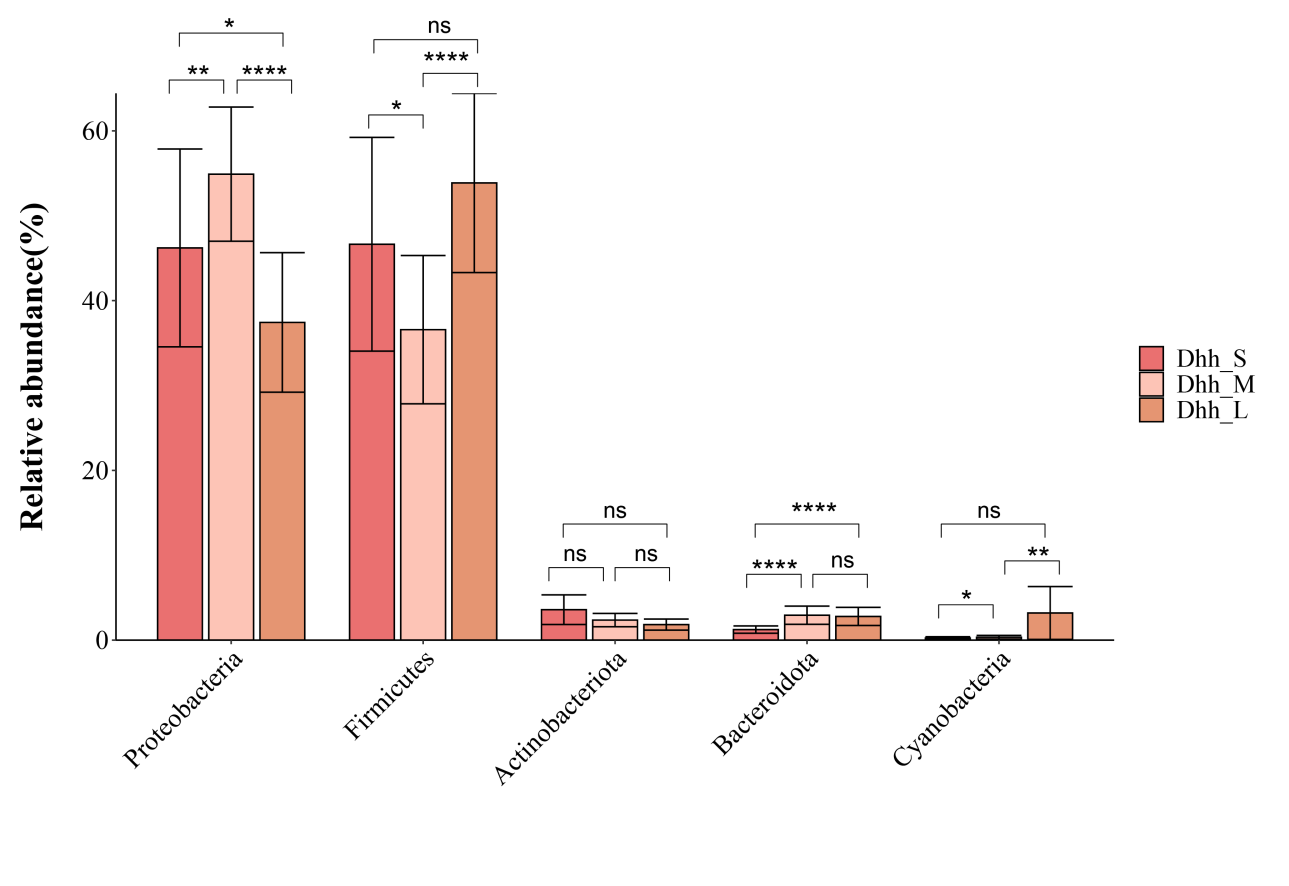

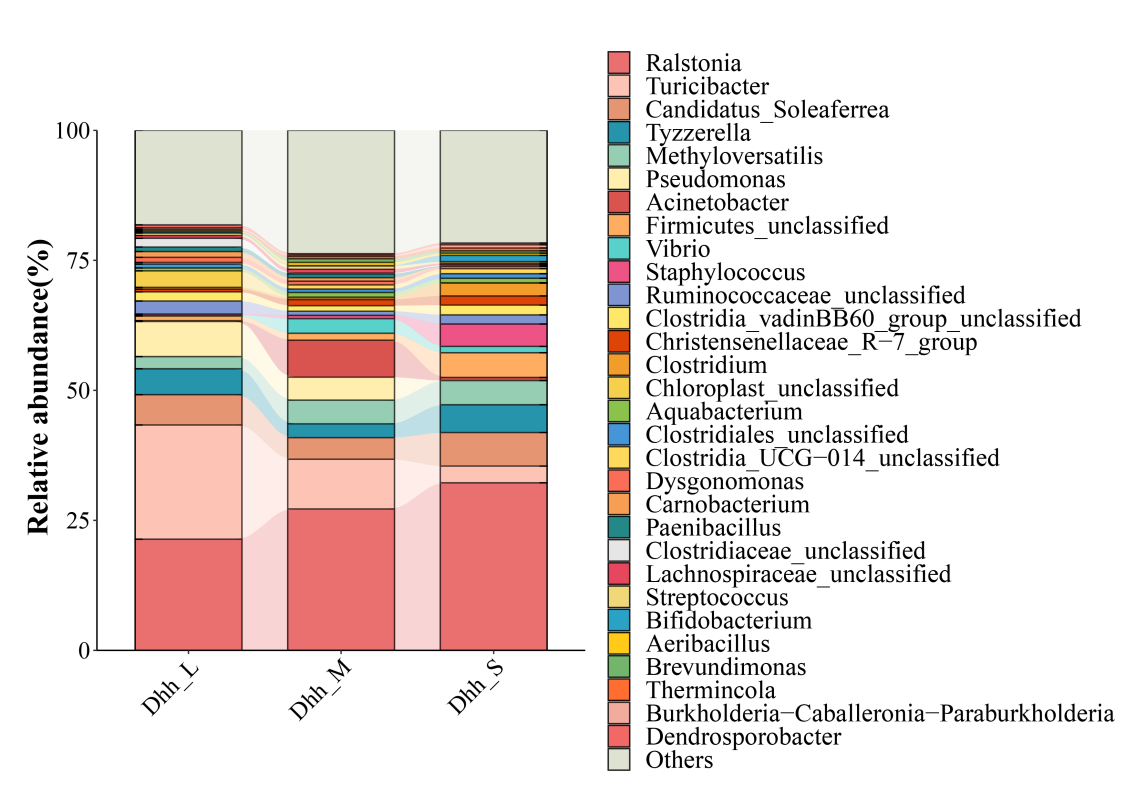


**A**

**B**

**Figure S3. The composition of genera among Dhh_M，Dhh_S and Dhh_L.** (A) The column diagram depicted the top 5 most abundance phyla difference. (B) The stacked plot demonstrated the top 30 most abundance bacterial genera distribution.


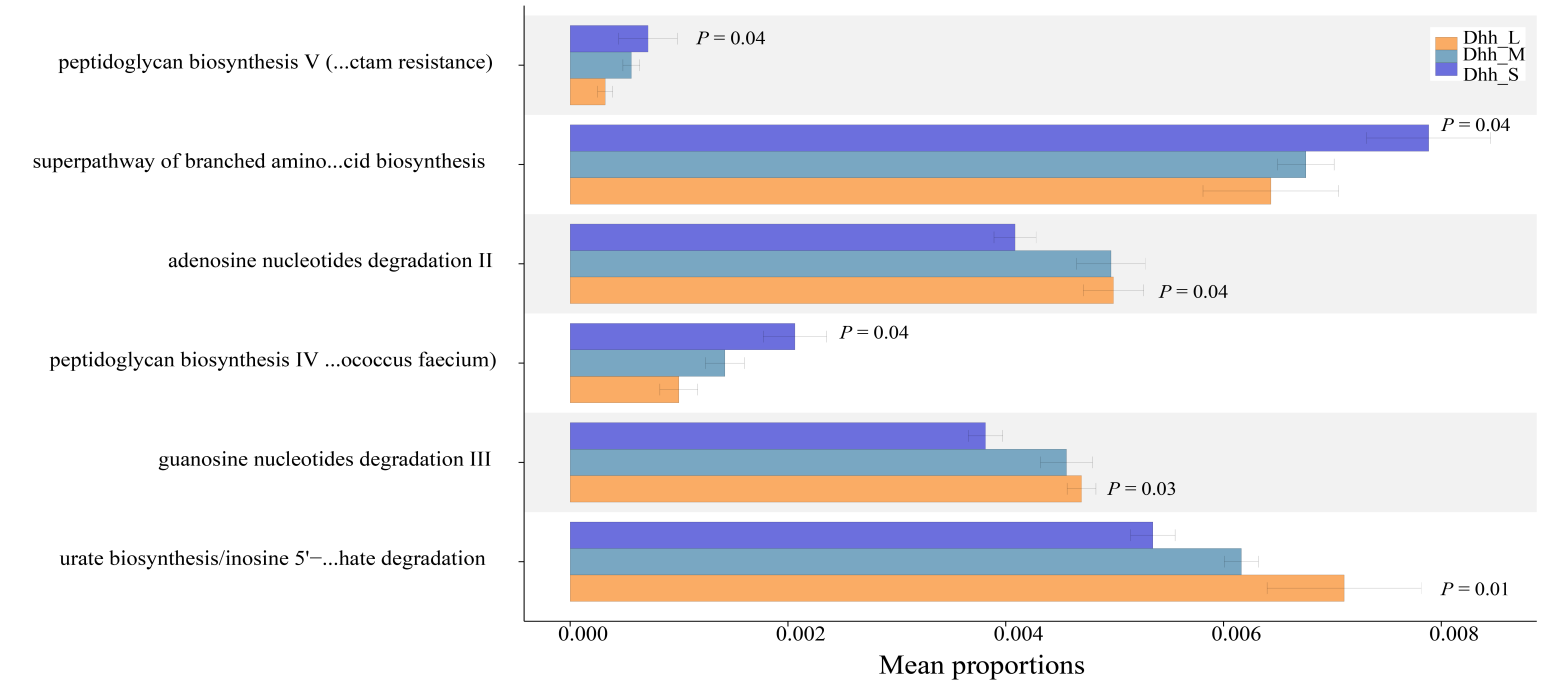

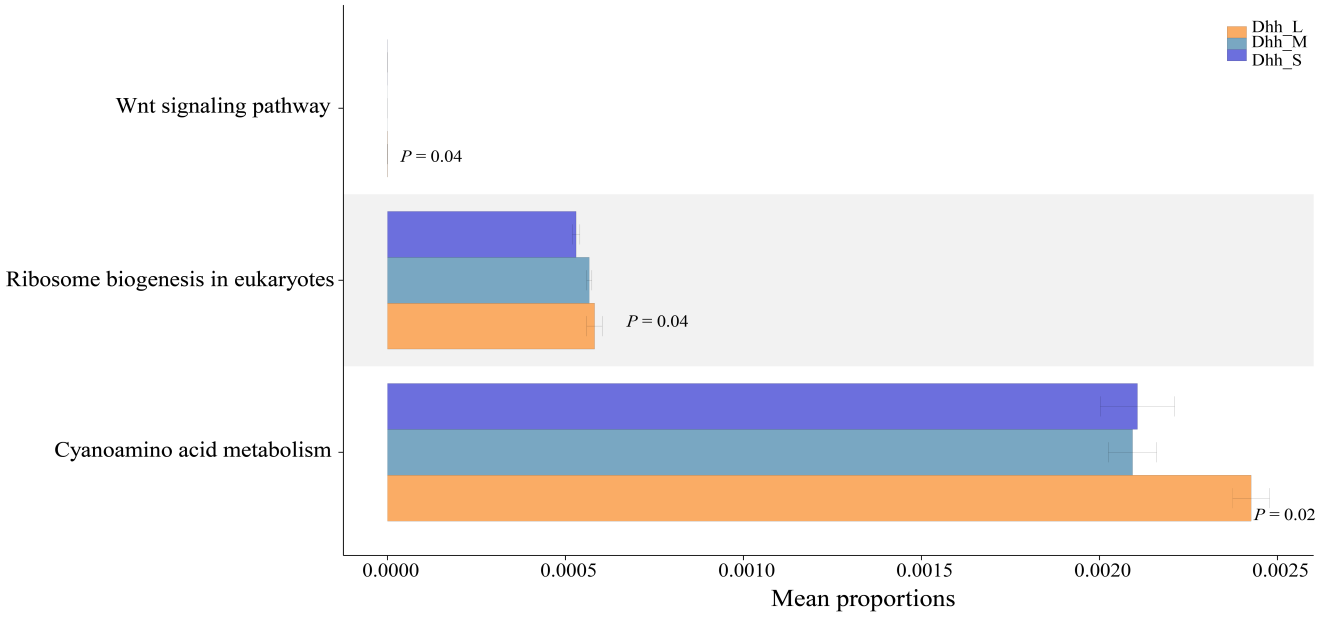


**A**

**B**


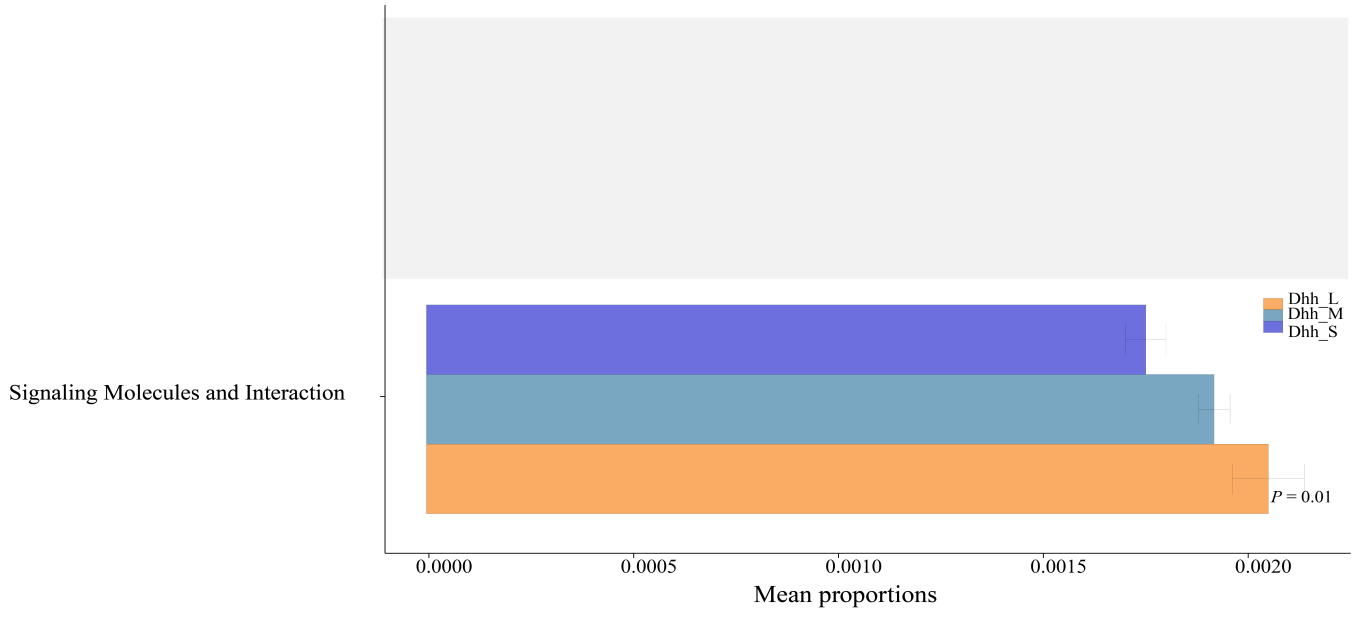


**C**

**Figure S4. Prediction of altered KEGG pathways using PICRUSt2 analysis of the fecal microbiota for groups Dhh_M，Dhh_S and Dhh_L.** The prediction of altered KEGG pathways in (A) level 2, (B) level 3 and (C) pathway. Bar plots on the left side display the mean proportion of each KEGG pathway. Dot plots on the right show the differences in mean proportions between the two indicated groups. P-value was calculated using t-test.

**Supplementary tables**

| **Table S1. Summary of gut microbiota sequencing information of wild and domestic** *Dorcus hopei hopei* | | | | | | | | | | |
| --- | --- | --- | --- | --- | --- | --- | --- | --- | --- | --- |
| **Sample** | **Raw_Tags** | **Raw_Bases** | **Valid_Tags** | **Valid_Bases** | **Valid%** | **Q20%** | **Q30%** | **GC%** | **Counts** | **ASV_number** |
| Dhh_D1 | 84812 | 42.41M | 73467 | 30.29M | 86.62 | 91.30 | 80.40 | 53.39 | 40372 | 209 |
| Dhh_D10 | 69037 | 34.52M | 53430 | 22.58M | 77.39 | 95.74 | 89.45 | 54.14 | 45338 | 146 |
| Dhh_D11 | 83237 | 41.62M | 74313 | 31.40M | 89.28 | 95.95 | 89.73 | 53.33 | 64667 | 299 |
| Dhh_D12 | 61070 | 30.54M | 49742 | 21.17M | 81.45 | 95.30 | 88.43 | 51.93 | 42071 | 102 |
| Dhh_D13 | 87220 | 43.61M | 80503 | 34.23M | 92.30 | 95.74 | 89.20 | 54.05 | 71077 | 206 |
| Dhh_D14 | 48490 | 24.25M | 42414 | 17.95M | 87.47 | 95.93 | 89.78 | 54.16 | 36152 | 142 |
| Dhh_D2 | 83566 | 41.78M | 73514 | 30.84M | 87.97 | 96.81 | 91.42 | 52.89 | 66852 | 74 |
| Dhh_D3 | 75021 | 37.51M | 63152 | 26.46M | 84.18 | 95.44 | 88.78 | 52.90 | 54036 | 106 |
| Dhh_D5 | 63897 | 31.95M | 57960 | 24.59M | 90.71 | 95.50 | 88.81 | 54.09 | 49393 | 225 |
| Dhh_D6 | 86454 | 43.23M | 80613 | 34.18M | 93.24 | 95.69 | 89.18 | 53.85 | 70537 | 208 |
| Dhh_D7 | 49376 | 24.69M | 44539 | 18.74M | 90.20 | 94.79 | 87.05 | 53.76 | 35651 | 221 |
| Dhh_D8 | 51993 | 26.00M | 44317 | 18.62M | 85.24 | 95.67 | 89.42 | 53.78 | 36370 | 319 |
| Dhh_D9 | 54100 | 27.05M | 48940 | 20.70M | 90.46 | 95.67 | 89.29 | 54.05 | 41158 | 304 |
| Dhh_W10 | 80614 | 40.31M | 72689 | 30.14M | 90.17 | 94.86 | 87.48 | 52.12 | 58475 | 387 |
| Dhh_W11 | 83606 | 41.80M | 75211 | 30.96M | 89.96 | 96.05 | 89.91 | 53.64 | 64845 | 848 |
| Dhh_W13 | 82205 | 41.10M | 73898 | 30.41M | 89.89 | 95.53 | 88.94 | 52.26 | 62242 | 772 |
| Dhh_W15 | 80764 | 40.38M | 71674 | 29.80M | 88.74 | 95.90 | 89.71 | 53.34 | 62534 | 281 |
| Dhh_W16 | 81554 | 40.78M | 67680 | 28.46M | 82.99 | 96.42 | 90.46 | 52.75 | 60917 | 378 |
| Dhh_W17 | 82634 | 41.32M | 75282 | 30.57M | 91.10 | 95.36 | 88.33 | 53.36 | 60672 | 811 |
| Dhh_W3 | 87446 | 43.72M | 78913 | 32.39M | 90.24 | 96.35 | 90.55 | 53.06 | 69403 | 392 |
| Dhh_W4 | 79638 | 39.82M | 71327 | 30.14M | 89.56 | 95.35 | 88.61 | 52.72 | 61053 | 330 |
| Dhh_W5 | 85254 | 42.63M | 77693 | 32.95M | 91.13 | 95.22 | 88.37 | 51.83 | 66993 | 291 |
| Dhh_W6 | 81515 | 40.76M | 69685 | 29.54M | 85.49 | 95.82 | 89.21 | 52.51 | 61489 | 278 |
| Dhh_W7 | 82333 | 41.17M | 73747 | 31.08M | 89.57 | 95.58 | 89.04 | 52.69 | 63677 | 301 |
| Dhh_W9 | 82545 | 41.27M | 72796 | 30.92M | 88.19 | 96.50 | 90.66 | 52.87 | 66206 | 266 |
| SUM | 1888381 | 944.22M | 1667599 | 699.11M | / | / | / | / | 1,412,180 | 4013 |
| AVG | 75535 | 37.77M | 66700 | 27.96M | 88.14 | 95.54 | 88.89 | 53.18 | 56,487.2 | 316 |

| **Table S2. Statistic analysis of alpha diversity indexs of gut microbiota among different sizes *Dhh* larval** | | | | |
| --- | --- | --- | --- | --- |
| P value | Dhh_M vs Dhh_S | Dhh_M vs Dhh_L | Dhh_L vs Dhh_S | Dhh_M vs Dhh_S vs Dhh_L |
| Chao_1 | 0.49 | 0.59 | 0.39 | 0.62 |
| Pielou | 0.56 | 0.83 | 0.78 | 0.84 |
| Simpson | 0.92 | 0.52 | 0.67 | 0.82 |
| Observed_otu | 0.49 | 0.74 | 0.39 | 0.65 |
| Goods_coverage | 0.77 | 0.16 | 0.2 | 0.3 |
